# 3D MR neurography with gadolinium contrast to improve the visualization of pelvic nerves and the branches

**DOI:** 10.3389/fphys.2024.1394431

**Published:** 2024-05-24

**Authors:** Hui Liu, Zhibo Xiao, Li Tao, Mingmei Tang, Yong Xu, Yuanrui Pan, Ke Zhang, Xueke Qiu, Fajin Lv

**Affiliations:** ^1^ State Key Laboratory of Ultrasound in Medicine and Engineering, College of Biomedical Engineering, Chongqing Medical University, Chongqing, China; ^2^ Department of Radiology, The First Affiliated Hospital of Chongqing Medical University, Chongqing, China

**Keywords:** magnetic resonance neurography, pelvic nerve, gadolinium, signal-to-noise ratio, contrast-to-noise ratio, contrast ratio

## Abstract

**Objective:**

To evaluate the effectiveness of 3D NerveVIEW sequence with gadolinium contrast on the visualization of pelvic nerves and their branches compared to that without contrast.

**Methods:**

Participants were scanned twice using 3D NerveVIEW sequence with and without gadolinium contrast to acquire pelvic nerve images. The signal-to-noise ratio (SNR), contrast-to-noise ratio (CNR) and contrast ratio of the nerves were calculated and compared to determine the quality of images. To subjectively assess, using a 3-point scale, branch nerves critical to therapeutic decision-making, including the pelvic splanchnic nerve and pelvic plexus, the superior gluteal nerve, and the pudendal nerve.

**Results:**

In the 32 eligible participants after using contrast, the CNRs of the images of nerve-to-bone and nerve-to-vessel significantly increased (*p* < 0.05). The CR of the images with contrast of all nerve-to-surrounding tissues (i.e., bone, muscle, blood vessels, and fat) were also found significantly higher (*p* < 0.05). The assessment of observers also shows higher scores for images with contrast compared to images without contrast.

**Conclusion:**

The 3D NerveVIEW sequence combined with gadolinium contrast improved vascular suppression, increased the contrast between pelvic nerves and surrounding tissue, and enhanced the visualization of nerves and their branches. This study may be helpful for the technically challenging preoperative planning of pelvic diseases surgery.

## 1 Introduction

The pelvic nervous system includes both somatic and autonomic nerves (Alkatout et al., 2021). The somatic nerves, originating from the lumbar and sacral spinal cord, directly innervate structures including the pelvic floor muscles, external urethral and anal sphincters, lower abdominal wall, and lower limbs (Alkatout et al., 2021). The autonomic nervous system, composed of sympathetic and parasympathetic systems, regulates the visceral functions of the pelvis and surrounding structures. The pelvic splanchnic nerves (PSN), emanating from the sacral nerve roots, innervate organs such as the rectum, anal canal, uterus, prostate, and bladder (Alkatout et al., 2021). In the treatment of benign or malignant pelvic tumors, inadequate intraoperative visualization of pelvic nerves can increase the risk of iatrogenic nerve damage, leading to severe voiding, anorectal, and sexual dysfunction (Bohrer et al., 2009; Puntambekar and Manchanda, 2018; Muallem et al., 2019; Alkatout et al., 2021). Additionally, surgeries such as hip replacement may cause injury to somatic nerves, particularly the smaller superior gluteal nerves, resulting in complications (Hasija et al., 2018). Therefore, precise preoperative visualization of these nerves is crucial in clinical practice to avoid potential intraoperative nerve injuries, ensuring the safety of the surgery and the functional recovery of patients.

Magnetic resonance imaging (MRI) is commonly used for examining pelvic diseases. Nowadays, preoperative scanning with enhancement is often performed to gain a comprehensive understanding of the location and nature of pelvic lesions, as well as the relationship between these lesions and nearby blood vessels. Magnetic resonance neuroimaging (MRN) is primarily employed to evaluate the associated nerves around the lesion. MRN sequences such as fat-suppressed T2-weighted imaging fast spin echo (T2WI-TSE) sequence and diffusion-weighted (DW-MRN) sequence are commonly used. However, these sequences have limitations. For instance, fat-suppressed T2WI-TSE sequences increase the minimum repetition time and acquisition time, while reducing the signal-to-noise ratio (SNR) (Tadenuma et al., 2020); Recently, an *ex-vivo* DWI (DTI) study of peripheral nerves using a high-field strength (9.4T) MRI and strong magnetic field gradient system clearly depicted the fascicular structure (fascicles, interfascicular epineurium, and even perineurium). However, in clinical *in vivo* MRI scanning, due to limitations such as magnetic field strength, magnetic field gradient, and scanning time, the resolution of DW-MRN is insufficient, making it difficult to accurately display the fine structure of the nerves (Pušnik et al., 2023). However, a novel MRN technique called the Three-dimensional (3D) NerveVIEW sequence has been developed to address these limitations. This technique combines a short tau inversion recovery (STIR) fat suppression technique with a set of equally sized diffusion-weighted prepulse of opposite polarity of action known as improved motion-sensitized driven equilibrium (iMSDE). The 3D NerveVIEW sequence enables higher-resolution imaging of peripheral nerves with improved signal-to-noise ratios and reasonable acquisition time. However, the visualization of the nerves and their branches is impeded by the slow-flowing vascular signals that run parallel to them. In particular, the visualization of the small pelvic nerves is inadequate (Yoneyama et al., 2013; Klupp et al., 2019).

The use of intravenous gadolinium contrast agents for improving peripheral neuroimaging has been explored. The post-gadolinium contrast 3D STIR-TSE sequence improved the nerve-to-muscle contrast ratio in both diseased and normal nerves, allowing for enhanced visualization and increased diagnostic confidence in the evaluation of the branch nerves of the brachial plexus (particularly the distal segments of the axillary nerve) (Wang et al., 2016; Sneag et al., 2020). However, it is currently unknown whether the enhanced 3D NerveVIEW sequence can improve pelvic vascular suppression and enhance the visualization of pelvic nerves and small branches.

The objective of this study was to evaluate the effectiveness of the enhanced 3D NerveVIEW sequence in improving pelvic vascular suppression, enhancing the contrast between pelvic nerves and surrounding tissues, and improving the visualization of nerves and their smaller branches.

## 2 Materials and methods

### 2.1 Participants

**FIGURE 1:**
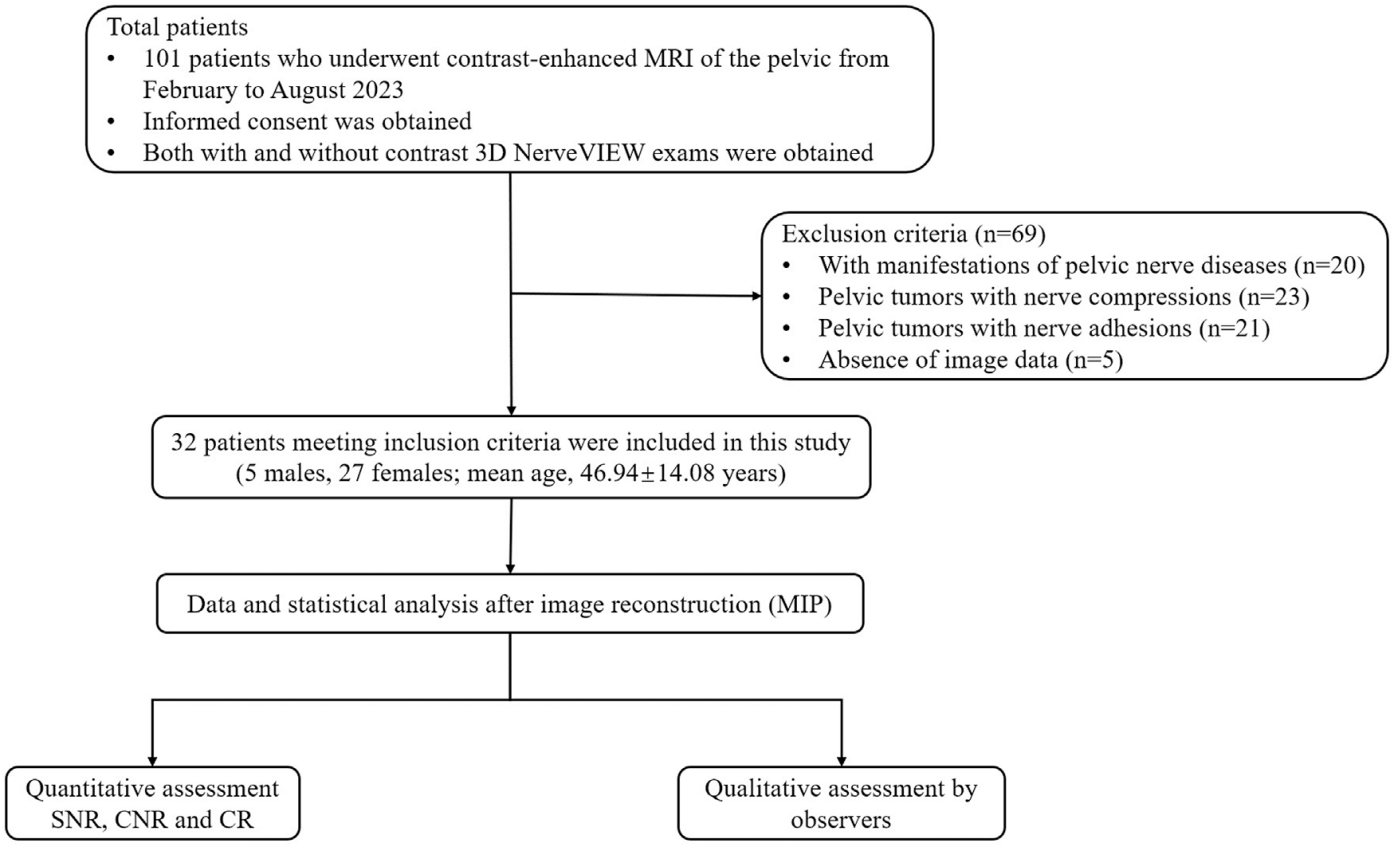
Flowchart of the participant selection. Abbreviations: MIP: maximum intensity projection; SNR: signal-to-noise ratio; CNR: contrast-to-noise ratio; CR: contrast ratio.

From February to August 2023, a total of 101 patients who underwent contrast-enhanced pelvic 3.0T magnetic resonance at our hospital were recruited in the study. Exclusion criteria included: Patients with manifestations of pelvic nerve diseases (n = 20) or pelvic tumors with nerve compressions (n = 23), pelvic tumors with nerve adhesions (n = 21), and absence of image data (n = 5). Finally, 32 patients were included in the study (Figure 1). This study was approved by the ethics committee of our hospital (Department of Radiology, The First Affiliated Hospital of Chongqing Medical University, a tertiary first-class general hospital), and all participants provided written informed consent.

### 2.2 MR examinations and sequence parameters

Each subject underwent routine MRI pre-examination preparation, which included weight measurement and the placement of intravenous indwelling needles. All examinations were conducted using a Philips Ingenia 3.0T MRI scanner (Philips Healthcare). Patients were asked to empty their bladder and bowel prior to MR examination. The subjects were positioned in a supine position with their hands raised above their heads. To obtain a high signal-to-noise ratio, a 32-channel abdominal coil (anterior coil) was utilized to cover the pelvis. The coil was positioned parallel to the coronal plane of the body and the scanning bed. Before and after gadolinium contrast administration, 3D NerveVIEW scans were performed. The contrast was injected by power injector (Medrad Spectris Solaris EP, Bayer Healthcare) at a flow rate of 2.0 mL/s and a dosage of 0.2 mmol/kg, followed by rinsing with 20 mL of physiological saline at the same injection rate.

The relevant parameters were optimized, including the iMSDE refocusing type, RF shims, flow gradient encoding (Venc), and fat suppression. The specific parameters used for the Coronal 3D NerveVIEW sequence, TR/TE/TI = 2,300/176/260 ms, field of view = 320 mm, Slice Thickness = 2 mm, Acquisition Voxel = 1.1 mm, NSA = 2, Number of Slices = 120, Pre-Pulse Preparation Time = 50 ms, Parallel imaging (SENSE) factors = P reduction (RL): 4, S reduction (AP): 1.5, matrix sizes = 292 × 292, Frequency-encoding direction = superior-inferior, Venc (RL, AP, FH) = 1 cm/s and total scan time of the sequence = 6:33 min.

### 2.3 Quantitative analysis

All sequences were analyzed on an IntelliSpace Portal post-processing workstation (NET framework version 1.1, Philips Healthcare, Netherlands). Objective quantification was performed by a radiologist with 3 years of experience in pelvic and peripheral nerve imaging. The nerves’ region of interest (ROI) was outlined bilaterally in the postganglionic segments of the 1st sacral nerve root on 15-mm coronal maximum intensity projection (MIP) images. This choice was made because the 1st sacral nerve is a larger branch of the pelvic somatic nerve, which allows for easier placement and measurement of the ROI. The ROIs were outlined while trying to avoid blood vessels, fat, and artifacts as much as possible. The area of the ROIs was approximately 15 mm^2^, and then a bilateral average was calculated as the final signal value for statistical analysis. Peripheral tissues such as perineural vessels, muscle, fat, and background air were selected while trying to minimize artifactual interference. The above measurements were performed twice, with a two-month interval, and the results were averaged to ensure data consistency. Then the SNR, contrast-to-noise ratio (CNR), and contrast ratio (CR) were calculated to assess image quality quantitatively according to the following equations ((Klupp et al., 2019; Welvaert and Rosseel, 2013)).

SNR = SI nerve/SD air.

CNR = (SI nerve—SI adjacent soft tissue)/SD air.

CR = (SI nerve–SI adjacent soft tissue)/(SI nerve + SI adjacent soft tissue).

### 2.4 Qualitative analysis

Images with and without contrast were independently reviewed by two radiologists with 7 and 11 years of experience in pelvic and peripheral nerve imaging, respectively. The radiologists were blinded to the clinical information of any patients and whether the sequence included Gd contrast. The evaluation of 3D NerveVIEW images before and after contrast administration was conducted using multiplanar reformation (MPR) and MIP (slice thickness = 3mm–20 mm) in a randomized order, respectively. Based on previous studies in obstetrics and gynecology, urology, and orthopedic (hip-related) surgeries that involved pelvic nerve injuries (Bohrer et al., 2009; Burge et al., 2014; Delaney et al., 2014; Yamashita et al., 2017), we identified the pelvic splanchnic nerves, pelvic plexus, superior gluteal nerves, and pudendal nerves as our target nerves. The quality of the bilateral nerve images was assessed using a 3-point grading scale, based on the presence of perineural artifacts, vascular interference, and the condition of the nerve trunk (Zhang et al., 2020), The grading scale was as follows: 2 = excellent, indicating good visualization without any vascular or artifactual interference; 1 = good, indicating moderate visualization with some vascular and artifactual interference; and 0 = poor, indicating poor visualization with severe vascular, artifactual, and noise issues.

### 2.5 Statistical analysis

Statistical analyses were conducted using SPSS version 26.0 (IBM Corp, 2019) and GraphPad Prism 9. Descriptive statistics were conducted after pooling of left and right sides as scored by the observers. The Shapiro-Wilk test was used to assess the normality of the data distribution. Continuous variables were reported as mean ± standard deviation (SD). Paired t-tests were employed to compare quantitative parameters, while Wilcoxon signed-rank tests were used for comparing qualitative parameters. Cohen’s kappa was used to measure the level of agreement between the two doctors (>0.81, excellent; 0.61–0.80, substantial; 0.41–0.60, moderate; 0.21–0.40, fair; and <0.20, poor). Statistical significance was set at a *p*-value of less than 0.05.

## 3 Results

### 3.1 Participant selection and characteristics

According to the inclusion and exclusion criteria, this study included 32 patients who did not show any involvement of adjacent neurovascular bundles, consisting of 5 males (15.63%) and 27 females (84.38%) (Figure 1). The mean age of the participants was 46.94(SD: 14.08) years. The sample contained 10 cases of uterine leiomyomas, 8 cases of ovarian tumors, 6 cases of cervical disease, 4 cases of rectal cancer, 2 cases each of endometrial disease and prostate cancer.

### 3.2 Quantitative analysis

**FIGURE 2:**
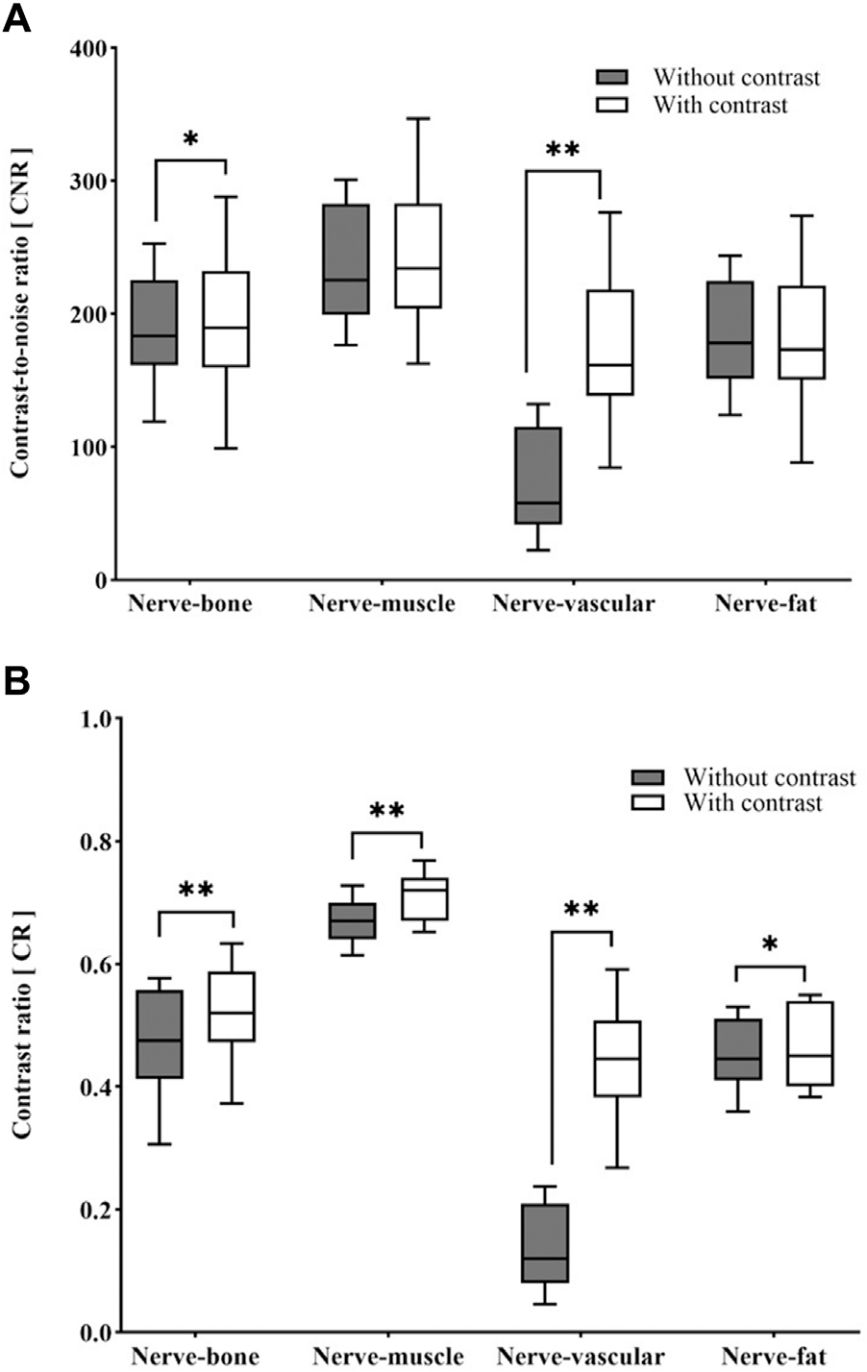
Box-and-whisker plots of the differences in CNR **(A)** and CR **(B)** between with and without contrast images. *p < 0.05, **p < 0.001. CNR: contrast-to-noise ratio; CR: contrast ratio.

Quantitative analysis showed that the SNR of nerves was higher on 3D NerveVIEW sequence images without contrast compared to that with contrast (301.89 ± 67.91 vs 295.35 ± 68.58; *p* < 0.005) (Table 1). After gadolinium contrast administration, the CNRs of nerve-vessel and nerve-bone were significantly improved (*p* < 0.05, *p* < 0.001). The CRs of nerves against surrounding tissues (i.e., fat, muscle, vein, and bone) were significantly higher (*p* < 0.05) in 3D NerveVIEW sequence images with contrast compared to those without contrast (Table 1; Figure 2).

**TABLE 1:** Comparison of quantitative analysis of 3D NerveVIEW sequence images with and without gadolinium contrast administration.

	Without Gd contrast	With Gd contrast	*p*-value
SNR			
S1	301.89 ± 67.91	295.35 ± 68.58	<0.05
CNR			
Nerve vs. bone	193. 11 ± 57.08	199.90 ± 59.47	<0.05
Nerve vs. muscle	242.83 ± 58.78	246.64 ± 62.30	0.076
Nerve vs. vein	79.93 ± 60.71	178.65 ± 58.56	<0.001
Nerve vs. fat	188.44 ± 55.33	188.25 ± 59.81	0.932
CR			
Nerve vs. bone	0.47 ± 0.10	0.51 ± 0.09	<0.001
Nerve vs. muscle	0.66 ± 0.02	0.70 ± 0.08	<0.001
Nerve vs. vein	0.15 ± 0.07	0.44 ± 0.11	<0.001
Nerve vs. fat	0.45 ± 0.07	0.46 ± 0.08	<0.05

SNR: signal-to-noise ratio; SN1: the 1st nerve; CNR: contrast-to-noise ratio; CR: contrast ratio.

### 3.3 Qualitative analysis

**FIGURE 3:**
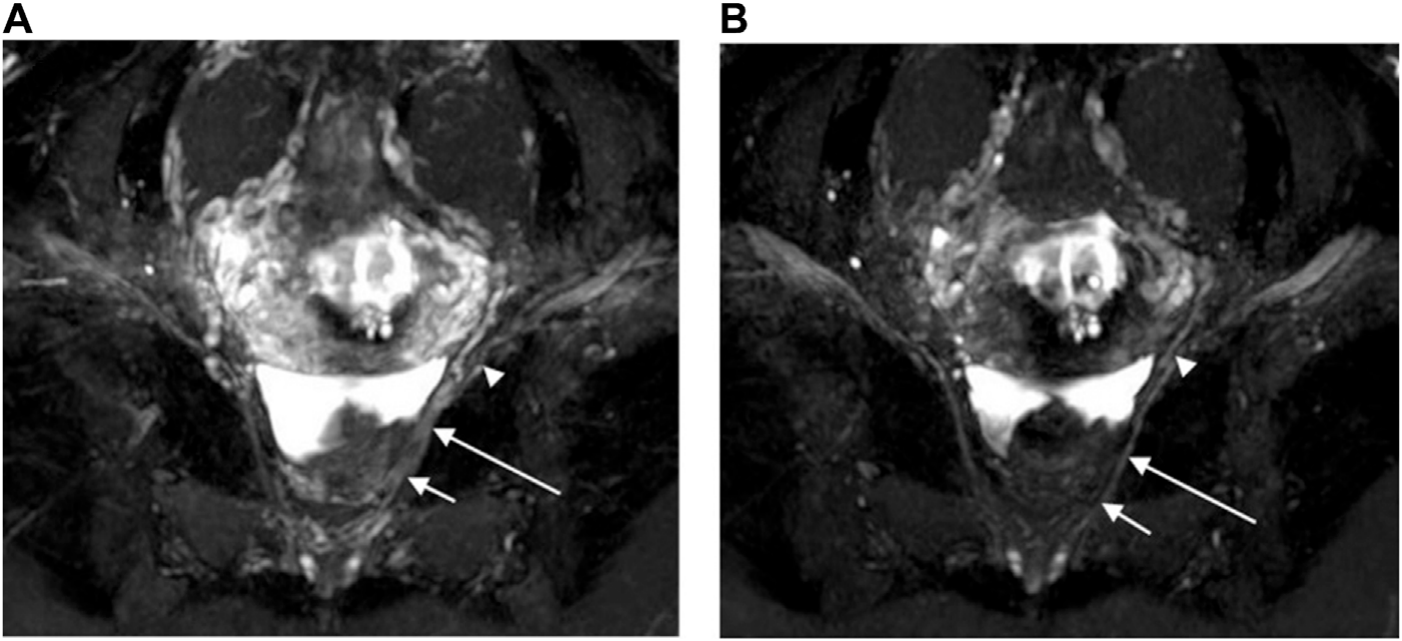
Oblique coronal MIP images (slice thickness = 15 mm) from a 3D NerveVIEW sequence were obtained for a 35-year-old female patient with a subserosal leiomyoma on the posterior wall of the uterus and a small amount of pelvic fluid. Image **(A)**, without gadolinium contrast, showed the nerves with unclear depiction due to overlapping adjacent tissues. However, image **(B)**, with gadolinium contrast, clearly demonstrated the pelvic splanchnic nerves (long arrows) extending from the sacral 3 nerve root (short arrows) to the pelvic plexus (arrowheads), without any overlap of vascular structures.

**FIGURE 4:**
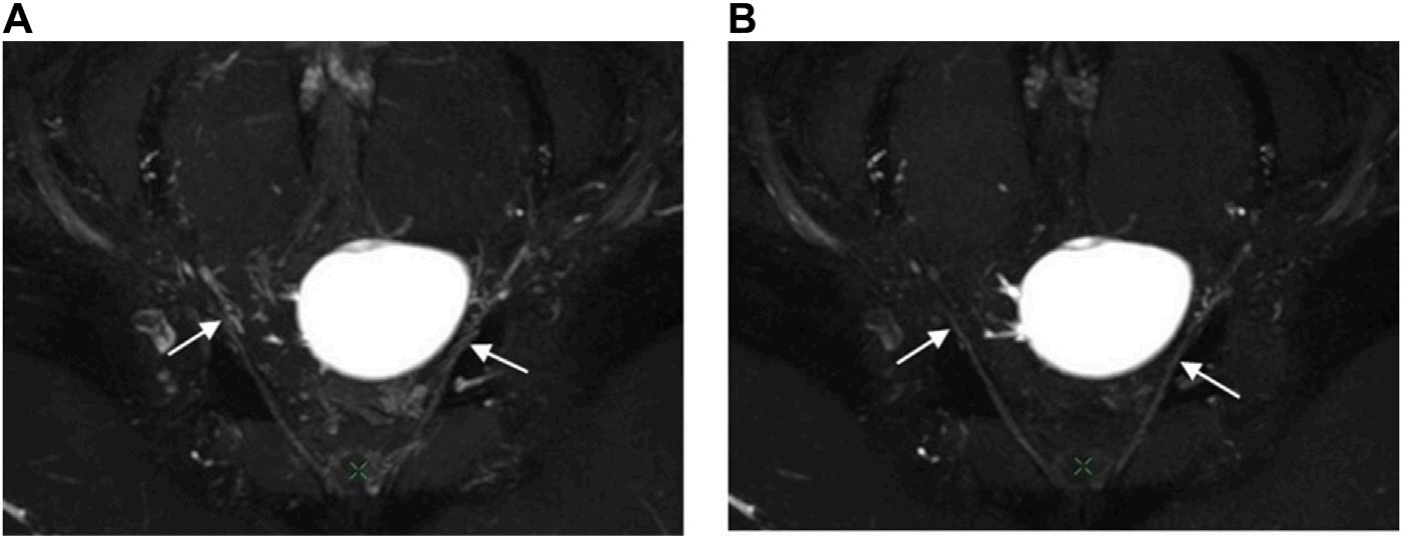
Oblique coronal MIP images (slice thickness = 15 mm) from a 3D NerveVIEW sequence were obtained for a 26-year-old female patient with a benign left ovarian mass. Image **(A)**, without gadolinium contrast, showed the pelvic splanchnic nerve (arrow) with unclear borders and discontinuous course due to interference from surrounding blood vessel signals. Image **(B)**, with gadolinium contrast, demonstrated a continuous and well-defined nerve (arrow) course as the gadolinium contrast suppressed the signals from the blood vessels surrounding the nerve.

**FIGURE 5:**
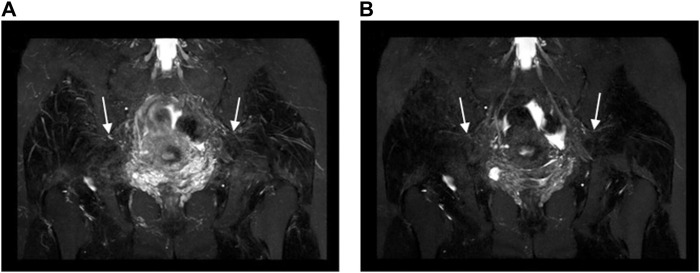
Oblique coronal MIP images (slice thickness = 15 mm) from a 3D NerveVIEW sequence were obtained for a 35-year-old female patient with a subserosal leiomyoma on the posterior wall of the uterus and a small amount of pelvic fluid. Image **(A)**, without gadolinium contrast, displayed the normal superior gluteal nerve with unclear borders and a discontinuous course, significantly affected by interference from surrounding blood vessel signals. Conversely, Image **(B)**, enhanced with gadolinium contrast, clearly demonstrated the normal superior gluteal nerve with distinct boundaries, a continuous course, and no interference from vascular signals.

**FIGURE 6:**
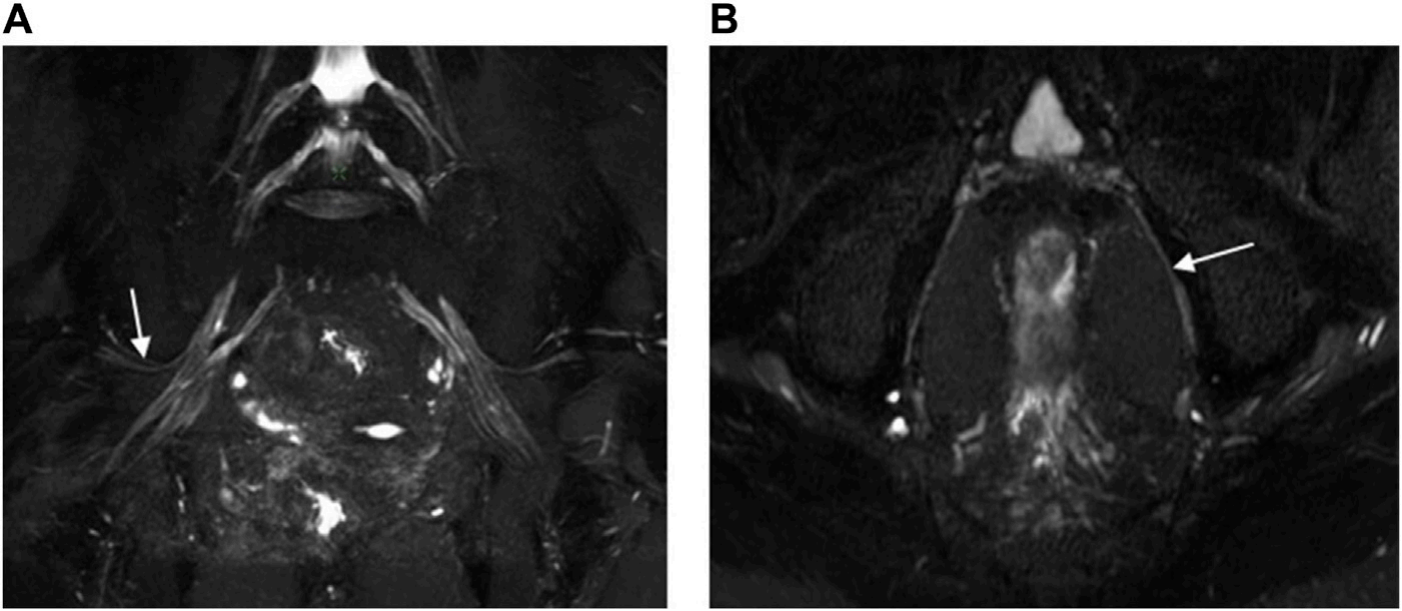
3D NerveVIEW sequence images of the superior gluteal nerves **(A)** and the pudendal nerves **(B)** were obtained using Gd contrast. Image **(A)** was an oblique coronal MIP view of the pelvis with a slice thickness of 15 mm for a 60-year-old female suspected of having cervical pathology. It displayed the normal superior gluteal nerve with clear boundaries, a continuous course, and no interference from vascular signals. Image **(B)** was an oblique axial MIP view of the pelvis with a slice thickness of 4 mm for a 60-year-old male with rectal cancer. It clearly delineated the boundaries of the pudendal nerve, showing a continuous course and no interference from vascular signals.

The scores regarding bilateral pelvic splanchnic nerves and pelvic plexus, superior gluteal nerves, and pudendal nerves were significantly higher (*p* < 0.01) in the 3D NerveVIEW sequence with contrast compared without contrast administration (Table 2; Figures 3–6). The agreement between the two radiologists were excellent for both the images with contrast (Kappa = 0.895–0.911) and those without contrast (Kappa = 0.821–0.894). Higher subjective scores indicate better visualization of the nerves and a better suppression of peripheral vascular and artifactual interference. Before contrast administration, the 3D NerveVIEW showed more vascular artifacts around the pelvic splanchnic nerves and pelvic plexus (Figure 3; Figure 4), as well as around the superior gluteal nerves (Figure 5), which affected the reliable identification of the nerves. However, after using gadolinium contrast, there was no interference around the nerves and the visualization ability was improved. Additionally, 3D NerveVIEW after contrast administration enhancement clearly showed the course of the superior gluteal and pudendal nerves (Figure 6).

**TABLE 2:** Comparison of scores for perineural vascularity and artifactual interference suppression in target nerves for unenhanced *versus* enhanced 3D NerveVIEW sequences.

	PSN	SGN	PN
with contrast	1.64 ± 0.57	1.55 ± 0.66	1.20 ± 0.76
without contrast	0.84 ± 0.62	0.67 ± 0.64	0.86 ± 0.59
Kappa	0.899	0.917	0.873

PSN: pelvic splanchnic nerves and pelvic plexus; SGN: superior gluteal nerves; PN: pudendal nerves.

## 4 Discussion

This study evaluated a new magnetic resonance neuroimaging sequence, the 3D NerveVIEW sequence, in combination with gadolinium contrast for imaging of the pelvic nerves. Results showed that the sequence, when combined with gadolinium contrast, effectively suppressed blood in displaying pelvic nerves and their branches, leading to higher subjective and objective image quality scores, and enhancing the visualization of pelvic nerves and branches. The use of post-processing techniques such as 3D MIP and MPR enabled multi-directional and multi-angle imaging, which are crucial for understanding the complex anatomical structure of the pelvic nerve network. In addition, optimization of parameters associated with the imaging quality of pelvic nerve 3D NerveVIEW sequence: fat suppression, RF shims, and flow velocity gradient encoding (Venc, equivalent to diffusion b-value), it was found that all pelvic nerve images obtained from the optimized 3D NerveVIEW sequence exhibited high signal-to-noise ratios and spatial resolution, with uniform suppression of fat and vascular signals and no artifactual interference, with or without the combination of a gadolinium contrast agent.

In this study, the STIR (Short TI Inversion Recovery) technique was employed as the fat suppression method. This inversion recovery technique suppressed fat signals at a designated inversion time, effectively enhancing the visualization of nerves by increasing the contrast with surrounding adipose tissue. Although STIR images exhibited a lower signal-to-noise ratio and more pulsation artifacts compared to other techniques such as frequency-selective adiabatic inversion recovery (SPAIR) (Kwee et al., 2014)and the iterative least squares algorithm for echoes of water-lipid separation (IDEAL) (Tomura et al., 2015), this technique proved applicable to all magnetic field strengths, was less susceptible to variations in B0 and B1 inhomogeneities, and was capable of performing fat suppression across a wide area of the pelvis. Moreover, STIR ensured uniform fat suppression and allowed for selective visualization of individual nerve plexus segments. Additionally, we used a high bandwidth (2500 HZ) inversion pulse of Nerve STIR to achieve robust fat suppression. In this study, we set the inversion time (TI) to 260 ms, slightly above the null point for fat at 3.0 T (which was approximately 205–225 ms) used in previous studies (Del Grande et al., 2014). This adjustment effectively enhanced fat suppression while preserving some background signal, facilitating the anatomical localization of nerves and lesions.

In pelvic 3.0T MRI scans, dielectric artifacts commonly occurred, leading to uneven image signal intensity and low central signals. To address this issue, we employed a Multi Transmit technique, which could be adaptively selected from the RF Shims option in the contrast parameters menu of the Philips 3.0T MRI scanner.

There are similarities between iMSDE and the concept of low b-value DWI, which is effective in suppressing physiological phenomena such as perfusion or turbulence in (semi)liquid substances within the body. However, there are also differences. iMSDE utilizes diffusion effects in pre-pulses with motion sensitization gradients, allowing for the free choice/design of the main sequence following the pre-pulse. Additionally, it can provide high-resolution images in a short acquisition time (Yoneyama et al., 2013; Yamashita et al., 2017). Appropriate iMSDE gradient strength (diffusion b-value) can suppress the vascular signal, here the diffusion b-value can be set by adjusting the magnitude of the Venc value, the lower the Venc value, the better the vascular suppression without distortion and blurring artifacts. Based on previous studies (Yoneyama et al., 2013; De et al., 2020), we used a low diffusion b-value technique, setting the Venc for the dimension-selective, phase-encoding, and frequency-encoding directions to 1 cm/s (equivalent to b = 10 s/mm^2^). This approach maximizes the dephasing of flowing blood without increasing the scanning time or affecting the resolution.

Previous studies had shown that intravenous administration of gadolinium contrast agents, when combined with 3D STIR-TSE, significantly improved vascular suppression in the brachial plexus, enhanced the contrast between nerves and surrounding tissues, and augmented the visualization of nerves and their branches, compared to non-contrast MRN sequences (Wang et al., 2016; Sneag et al., 2020). Our findings were consistent with these studies, and non-contrast MRN sequences faced challenges in differentiating nerves from adjacent blood vessels due to their similar relaxivity values and morphology (Does and Snyder, 1995). Despite employing the iMSDE pulse within the 3D NerveVIEW sequence, its efficacy in suppressing slow-flowing blood signals in the pelvis was limited. This limitation arose because the motion-sensitive gradients relied on effectively dephasing fast-flowing spins without impacting slow-flowing ones. Consequently, these slow blood flows hindered the assessment of clinically relevant small peripheral nerves (Does and Snyder, 1995; Yoneyama et al., 2013; Sneag et al., 2020; Queler et al., 2021). Nevertheless, due to the gadolinium contrast agents’ ability to shorten the T1 and T2 signals of blood, they could effectively suppress signals from vessels with small diameters or slow blood flow. This suppression of background signals further enhanced the CR between the nerves and the background. (Pedrick et al., 2023).

Objective metrics such as CR and CNR were crucial for evaluating nerve visualization. Higher values of these metrics correlated with enhanced grayscale performance in images, thus improving image readability and interpretability (Zhang et al., 2020). Furthermore, the 3D NerveVIEW sequence with intravenous gadolinium contrast demonstrated higher subjective scores in visualizing pelvic nerves (such as pelvic splanchnic nerves, superior gluteal nerves, and pudendal nerves) compared to the non-contrast method. This further substantiated the role of contrast-enhanced 3D NerveVIEW sequences in improving the visualization of pelvic nerves and their branches, particularly excelling at clearly depicting nerves not involved by pelvic tumors. However, when this technique failed to visualize nerves within tumor-affected areas, it raised concerns about potential nerve damage, an issue our technology specifically addressed. Additionally, the anatomical layout of pelvic nerves usually exhibited bilateral symmetry, which aided in identifying abnormalities through comparison. In cases where a malignant tumor affected only one side, the normal appearance of nerves in the contralateral healthy area further confirmed the involvement of nerves on the tumor-affected side.

Additionally, our imaging technology had significantly enhanced the visualization of pelvic nerves, enabling the mapping of entire neural regions by delineating areas of interest. This advancement facilitated both manual and automated software-based nerve segmentation (Sveinsson et al., 2022). Such an approach provided detailed anatomical pathways of nerves surrounding lesions, aiding surgeons in making preoperative decisions about nerve preservation and achieving precise intraoperative localization. Therefore, we believed our method held substantial clinical utility in assessing, predicting, and maintaining functional outcomes following nerve-sparing surgeries.

This study had several limitations. Firstly, we did not assess the optimal imaging time and contrast dose. The contrast dose we used was based on the routine dose used in clinical MRI enhancement examinations for pelvic diseases, and the optimal imaging time after contrast administration was performed after the end of the routine clinical pelvic diseases enhancement program to maximize the arrival of contrast into the small veins. Secondly, there were no clear criteria for determining the tilt angle of post-processed MIP images during subjective and objective assessment. Therefore, the ability to successfully depict the pelvic nerves might vary between imaging centers due to anatomical differences between subjects. Additionally, this method will still need to be combined with routine T1WI and T2WI sequences for clinical diagnosis.

## 5 Conclusion

In conclusion, enhanced 3D NerveVIEW is a powerful vascular suppression technique that significantly improves pelvic nerve contrast with surrounding tissue and enhances visualization of fine branches. This may provide a more detailed anatomical roadmap for preoperative planning regarding pelvic surgery.

## Data Availability

The raw data supporting the conclusion of this article will be made available by the authors, without undue reservation.
